# Recreational Screen Time and Anxiety among College Athletes: Findings from Shanghai

**DOI:** 10.3390/ijerph18147470

**Published:** 2021-07-13

**Authors:** Yu Gao, Ning Fu, Yuping Mao, Lu Shi

**Affiliations:** 1 Department of Physical Education, Shanghai University of Finance and Economics, 111 Wuchuan Rd, Yangpu District, Shanghai 200434, China; gaoyu@mail.shufe.edu.cn; 2 School of Economics, Shanghai University of Finance and Economics, 111 Wuchuan Rd, Yangpu District, Shanghai 200434, China; 3 Department of Communication Studies, California State University, Long Beach, CA 90840, USA; Yuping.Mao@csulb.edu; 4 Department of Public Health Sciences, Clemson University, Clemson, SC 29634, USA; LUS@clemson.edu

**Keywords:** college athlete, recreational screen time, anxiety, dispositional anxiety, situational anxiety

## Abstract

To better understand the behavioral factors contributing to the mental health status among student athletes, we examined the link between recreational screen time and college student athlete’s anxieties. This cross-sectional study was conducted among 278 college student athletes from Shanghai, China, aged between 17 and 25 years old (*M* = 19.4, *SD* = 1.5). Multivariate regression analyses, controlled for age, gender, rural vs. urban residency, and individual vs. team sports factors, were performed to analyze the association between their average daily recreational screen time in a week and their dispositional anxiety, pre-competition anxiety, and anxiety during competition, which were measured by the Chinese version of validated psychometric scales among athlete population. Significant results were found in both dispositional anxiety and situational anxiety in relation to recreational screen time among college athletes. Conclusions: Our findings indicate that excessive recreational screen time is a risk indicator of college student athletes’ dispositional anxiety, pre-competition anxiety, and anxiety during competition.

## 1. Background

Despite presumably high levels of physical activity and physical fitness, college student athletes face many health challenges in their young adulthood [1]. Aside from sports injuries [2] and sports-related chronic health issues (such as irregular menstrual cycles among female college athletes [3]), student athletes face excessive psychological burden of balancing between sports performance and academic endeavors and are more susceptible to mental health issues such as stress and depression [4]. Numerous studies conducted in different countries yielded consistent results that elite athletes experience a higher risk of mental disorders such as anxiety and depression [5]. However, there is a lack of epidemiological studies on risk factors for mental disorders among this population, and there is limited intervention-based research on student athletes’ mental health issues [5].

A plethora of literature has studied anxiety in sport psychology [6]. Anxiety, as feelings of nervousness, uneasiness, and tension about performance, can be categorized into dispositional anxiety and situational anxiety [7]. Dispositional anxiety is based on individual trait differences, and situational anxiety is influenced by environmental and situational factors [8]. Student athletes are subjective to both dispositional and situational anxieties, and they are constantly experiencing situational anxieties before and during competitions. Common stressors among athletes include injury, poor performance, fatigue, and organizational factors [5]. College student athletes with anxiety symptoms face higher risks of injuries [9,10]. Many existing studies have identified anxiety as a factor negatively affecting workplace performance [11]. Some research has found that sports experience was negatively related to anxiety [12].

There has been evidence that interventions aimed at reducing anxiety among college students, such as expressive writing [13] and reappraising psychological arousal [14], significantly improved their scholastic test performance [15], which is very important for a timely completion of the degree program for student athletes. Athletes use a wide range of strategies to cope with stress and anxiety such as goal-setting, mental preparation, or progressive relaxation training. These interventions were found to be effective in reducing student athletes’ anxiety and enhance sports performance [16,17,18].

Giving the rising popularity of electronic devices in recent decades [19,20], it is important to study the possible association between screen time and anxiety among student athletes. According to the uses and gratifications theory, individuals use media to serve different needs including surveillance, information learning, entertainment, personal identity, companionship, and escape [21,22]. Student athletes could use screen time to reduce their anxiety. While screen time has been found to be a risk factor for anxiety, the specific amount of screen time and its impact on anxiety has been inconclusive [23]. There has been evidence that extensive use of recreational screen time is associated with higher levels of anxiety and unhealthy behaviors among adolescents [24]. Few studies (if any) have examined the possible link between anxiety and screen time among college student athletes, especially their competition-related anxiety. In this study, we examine the link between recreational screen time and student athletes’ dispositional anxiety and situational anxiety (pre-competition and competition anxiety). Findings from this research can be used to provide insights for those student athletes with high levels of anxiety to manage their anxiety levels.

## 2. Method

This study is a cross-sectional analysis of baseline data from a randomized controlled trial of an anxiety reduction program using mindfulness practices among college student athletes in Shanghai, China. A total of 290 student athletes were recruited on a voluntary basis and filled out the baseline questionnaire about their mental health status and health behavior. The final analysis was carried out on a sample of 278 participants, including both men (36%) and women (64%), aged between 17 and 25 (M = 19.4; SD = 1.5). Students practiced individual sports (73%) including badminton, bridge, chess, fencing, golf, ping pong, skipping rope, swimming, taekwondo, and tennis, and some practiced team sports (27%) including soccer, volleyball, cheerleading, and basketball. Twelve participants were excluded because of missing data on demographic characteristics, recreational screen time, or anxiety scales. Specific information on the characteristics of the sample can be found in Table 1.

In the questionnaire, respondents reported their average daily recreational screen time in the previous week from the time of taking the questionnaire, by choosing one the following five options: (1) none; (2) less than 2 h; (3) 2–3 h; (4) 3–5 h; (5) more than 5 h. Participants were asked to select appropriate responses to indicate how they felt at the very moment of filling out the questionnaire when there were no games.

Participants’ levels of dispositional anxiety, pre-competition, and anxiety during competition were measured by a Chinese version of three validated psychometric scales among the athlete population [25]: “Athlete Trait Anxiety Scale”, “Athlete State Anxiety Scale (before competition)”, and “Athlete State Anxiety Scale (in competition)”. Three instruments comprised a total of 28 items rated using a Likert scale with 5 options ranging from 1 (strongly disagree) to 5 (strongly agree). See the questionnaire questions in Appendix A. Pre-competition anxiety and anxiety during competition were recall-based. These scales were based on Spielberger’s State Anxiety Inventory (SAI) [26] and Sport Competition Anxiety Test (SCAT) [27] and were adopted to the Chinese athlete population. They were chosen based on validity and reliability. All scales had adequate construct validity and reliability to assess mental health in sports. Wu and Lin (2009) reported Cronbach’s α values of each of the three scales: 0.8682 for athlete trait anxiety, 0.8333 for pre-competition anxiety, and 0.8483 for anxiety during competition. In our data, the Cronbach’s α was 0.923 for anxiety of trait, 0.9055 for pre-competition anxiety, and 0.8611 for anxiety during competition. The reliability coefficient for each scale was high, indicating the questionnaires were reliable.

Three multivariate regression analyses with robust standard errors, adjusting the covariates of age, gender, rural or urban residency, and individual vs. team sports, were performed to examine the link between recreational screen time (“none” as the referent group) and the three anxiety outcomes. STATA 14.0 MP (StataCorp, College Station, TX, USA) was used to analyzed data.

The study was approved by the office of research at Shanghai University of Finance and Economics.

## 3. Results

On average, the participants spent 3.9 h on recreation screen time. Specifically, 1.1% reported no recreation screen time, 13.3% less than 2 h, 24.1% between 2 and 3 h, 13.9% between 3 and 5 h, and 41.8% more than 5 h. Participants reported similar levels of dispositional anxiety (M = 2.2, SD = 0.77), pre-competition anxiety (M = 1.8, SD = 0.69), and anxiety during competition (M = 2.04, SD = 0.661), see Table 2. It is also worth noting that a small percentage of participants reported high levels of dispositional anxiety (13%) and recalled high levels of pre-competition anxiety (6%) and anxiety during competition (5.4%).

The Spearman rank correlation coefficients between the variable of recreational screen time and each of other covariates were all very low (less than 0.05) and were not significant at the 5% level as presented in Table A1 and Table A2. There was no indicator of multicollinearity. The univariate normality of outcome variables was supported since the absolute value of each scale’s skewness was below 2 (ranged from 0.503 to 1.338) and kurtosis was below 7 (ranged from 3.419 to 4.448). Table 2 provides detailed information on the distribution of scales.

There were significant findings on both dispositional anxiety and situational anxiety (pre-competition anxiety) in relation to recreational media use among student athletes in our study as reported in Table 3. In comparison with the non-recreational screen time group, student athletes with all four levels of recreational screen time were found to have significantly higher levels of dispositional anxiety. Student athletes with 3–5 h per day of recreational screen time and those with more than five hours of recreational screen time reported significantly higher levels of pre-competition anxiety than those with no recreational screen time. There were no significant associations found between student athlete anxiety with age, gender, urban/rural residency, or individual vs. team sports.

## 4. Discussion

Our findings indicate that excessive recreational screen time is a risk factor for student athletes’ dispositional anxiety, pre-competition anxiety, and during-competition anxiety. This is consistent with previous findings that more time spent on social media is associated with depression, self-harm, and lower level of self-esteem [28]. The “reverse causality” interpretation, however, could also be plausible: student athletes with higher levels of anxiety are more likely to use recreational screen time to cope with their anxiety. If further studies establish the causal link between recreational screen time and anxiety disorders, interventions can be designed and introduced to reduce and/or replace recreational screen time to help student athletes manage their anxieties.

From the perspective of intervention, the association between heavy recreational media use and situational anxiety shown in our study suggests the potential of designing and applying featured mobile phone applications to help athletes effectively cope with pre-competition and competition anxiety. A meta-analysis of 34 studies indicated that psychological interventions had small to medium-sized effects on competitive anxiety in athletes, and the effects were greater for athletes of higher levels of competition than those from lower levels of competition [29]. Existing sport anxiety intervention programs have included mental skill techniques such as self-talk, imagery, pre-performance routines, goal setting, cognitive restructuring, and relaxation techniques. More recently, mindfulness [30] and biofeedback [31] training have gained more prominence in sport anxiety training programs. As our study shows association between screen time and student athletes’ dispositional and situational anxiety, future interventions might consider incorporating effective trainings into interactive programs on mobile phones or computers that athletes with high anxiety levels already spend a lot of time on. Utilizing electronic media as a new platform of delivering anxiety intervention programs could make them more convenient, acceptable, and easier for student athletes to access.

Our study does not provide information on the competition performance of the five groups of athletes with different lengths of screen time. A meta-analysis of early research indicates the negative relationship between anxiety and performance [32]. Theories in sport psychology indicate the possibility for sports anxiety to benefit competition performance [33]. Empirical evidence shows perceiving anxiety facilitates performance and is associated with higher performance [34]. In addition, research has found a positive relationship between situational anxiety and rock-climbing performance for experienced rock climbers [35]. Recent theoretical developments in workplace anxiety also pinpoint factors such as motivation, ability, and emotional intelligence can play a critical role on whether anxiety will debilitate or facilitate job performance [11]. For student athletes, a certain amount of anxiety in combination with other factors could positively affect their competition performance. Therefore, intervention programs might not need to aim at reducing anxiety levels of all student athletes but should focus on those with excessive screen time and high anxiety levels.

While our study provided the above-mentioned findings, a few limitations need to be considered when applying the results in other contexts or designing future research. First, this study is based on a nonprobability sample from Shanghai. Student athletes from different social and cultural contexts could show different associational patterns when it comes to the link between recreational screen time use and anxiety disorders. Second, the student athlete sample could be very different from professional athletes since those two types of athletes have different expectations and different levels of stress of their performances in competitions. This college athlete population is also different from non-athlete college students in their health behavior. Third, our study had a limited number of covariates (only age, gender, urban/rural residential status, and individual vs. team sports). Future research should consider including more variables that could be related to anxiety level, such as years of experience in competitive sports and different categories of sports [18]. Sleep quality and sleep duration before competitions are also important measures to investigate as the relationship between screen time and suboptimal mental health could also be mediated by inadequate sleep [36]. Fourth, when the questionnaires were taken, there were no competitions or tournaments, only regular trainings; therefore, the measurement of their screen time relies primarily on participants’ recall at the time of filling out the questionnaires. This might not be the most accurate and objective way of measuring screen time since individuals may underestimate or overestimate their time for various reasons. A better approach, if future research budget permits, could be to ask the study participants to keep a time use diary (TUD) of their daily screen time [28].

## 5. Conclusions

Our research found that excessive recreational screen time is a risk factor for student athletes’ dispositional anxiety, pre-competition anxiety, and during-competition anxiety. In comparison with the non-recreational screen time group, student athletes with all four levels of recreational screen time (less than 2 h, between 2 and 3 h, between 3 and 5 h, and more than 5 h were found to have significantly higher levels of dispositional anxiety. Student athletes with 3–5 h per day of recreational screen time and those with more than five hours of recreational screen time reported significantly higher levels of pre-competition anxiety than those with no recreational screen time. Our findings indicate that excessive recreational screen time is a risk factor for student athletes’ dispositional anxiety, pre-competition anxiety, and during-competition anxiety. Our findings provide insights on the potential of designing and implementing of anxiety intervention programs via electronic media to help student athletes being negatively impacted by their high level of anxiety to manage their mental health and improve their sport and academic performances.

## Figures and Tables

**Table 1:** Sample Characteristics Data are *n* (%) or Mean (sd). *N* = 278.

Characteristics	Mean (SD)/*N* (%)	Min	Max
Age	19.4 (1.5)	17	25
Female	101 (36%)		
Male	177 (64%)		
Urban Residency	228 (82%)		
Rural Residency	50 (18%)		
Team Sports	76 (27%)		
Soccer	26 (35%)		
Volleyball	13 (17%)		
Cheerleading	18 (23%)		
Basketball	18 (23%)		
Individual Sports	202 (73%)		
Aerobics	67 (33%)		
Athletics	13 (6.4%)		
Badminton	9 (4.5%)		
Bridge	8 (4%)		
Chess	15 (7.4%)		
Fencing	10 (5%)		
Golf	4 (2%)		
Ping Pong	12 (6%)		
Skipping rope	22 (11%)		
Swimming	13 (6.4%)		
Taekwondo	10 (5%)		
Tennis	6 (3%)		

**Table 2:** The distribution of dependent variables.

Dependent Variables	M	SD	Skewness	Kurtosis
Anxiety	2.21	0.767	0.503	3.419
Pre-competition anxiety	1.84	0.686	1.038	4.448
Anxiety during competition	2.036	0.661	0.585	3.558

**Table 3:** Regression results on anxiety levels and recreational screen time among college athletes.

	Anxiety			Pre-Competition Anxiety			Anxiety during Competition		
	B	SE	*p*-Value	B	SE	*p*-Value	B	SE	*p*-Value
Recreational Screen Time ^1^									
less than 2 h	0.91 **	0.287	0.002	0.57 **	0.200	0.005	0.63 **	0.211	0.003
2–3 h	0.78 **	0.276	0.005	0.54 **	0.189	0.005	0.55 **	0.198	0.006
3–5 h	0.92 **	0.267	0.001	0.64 **	0.188	0.001	0.67 **	0.202	0.001
more than 5 h	0.80 **	0.263	0.003	0.58 **	0.174	0.001	0.57 **	0.188	0.003
Age	−0.01	0.033	0.789	0.00	0.030	0.985	0.00	0.028	0.918
Gender, female [ref, male]	0.07	0.102	0.494	0.01	0.096	0.9	−0.04	0.091	0.637
Urban, [ref, rural]	−0.07	0.116	0.556	−0.02	0.101	0.86	0.10	0.099	0.305
Group, [ref, individual]	−0.01	0.111	0.897	−0.06	0.095	0.524	0.01	0.094	0.882
Intercept	1.57	0.675	0.021	1.28	0.605	0.036	1.33	0.569	0.02
*N*	278			275 ^2^			278		

Note: ^1^. “No recreational screen time” was used as the reference group. ^2^. Three participants were missing pre-competition anxiety data and were not included in the pre-competition anxiety regression analysis ** *p* < 0.005.

## Data Availability

The data presented in this study are available on request from the corresponding author.

## Appendix A. Measures and Instruments

	**Athlete Trait Anxiety Scale**					
		**Strongly Disagree**	**Disagree**	**Neutral**	**Agree**	**Strongly Agree**
1	You feel nervous unconsciously.	1	2	3	4	5
2	You get nervous when facing a challenging job.	1	2	3	4	5
3	You feel frustrated when you encounter difficulties.	1	2	3	4	5
4	You often feel anxious when doing things.	1	2	3	4	5
5	You feel inferior to others.	1	2	3	4	5
6	You worry about your performance.	1	2	3	4	5
7	You often can’t concentrate.	1	2	3	4	5
8	You will feel anxious when you think of failure.	1	2	3	4	5
9	You feel uncomfortable in front of everyone.	1	2	3	4	5
	**Athlete State Anxiety Scale (before Competition)**					
		**Strongly Disagree**	**Disagree**	**Neutral**	**Agree**	**Strongly Agree**
1	You will feel trembling in your hands and feet before the game.	1	2	3	4	5
2	You will turn pale before the game	1	2	3	4	5
3	You will feel nauseous before the game	1	2	3	4	5
4	You will lose concentration before the game	1	2	3	4	5
5	You will feel a trance before the game	1	2	3	4	5
6	You will be afraid of the game before the game	1	2	3	4	5
7	You will feel dry mouth and tongue before the game	1	2	3	4	5
8	You will be afraid of making mistakes before the game	1	2	3	4	5
9	You will worry about bad performance before the game	1	2	3	4	5
						
	**Athlete Status Scale (in Competition)**					
		**Strongly Disagree**	**Disagree**	**Neutral**	**Agree**	**Strongly Agree**
1	You will feel nervous during the game.	1	2	3	4	5
2	You will feel trembling hands and feet during the game.	1	2	3	4	5
3	You will feel stage fright during the game.	1	2	3	4	5
4	You will feel tight muscles during the game.	1	2	3	4	5
5	Your palms sweat easily during the game.	1	2	3	4	5
6	You will feel relaxed during the game.	1	2	3	4	5
7	You will lose your temper easily during the game.	1	2	3	4	5
8	You will feel irritable during the game.	1	2	3	4	5
9	You feel anxious when you fall behind in the game.	1	2	3	4	5
10	You will feel uneasy when entering a tug of war during a game.	1	2	3	4	5

## Appendix B. Correlation Matrix of Regression Variables

**Table A1:** Spearman correlation matrix (recreation screen time as an ordinal variable).

	Anxiety	Preanxiety	Midanxiety	Screen	Age	Female	Urban	Group
**anxiety**	1							
**preanxiety**	0.6764 *	1						
**midanxiety**	0.5908 *	0.7126 *	1					
**screen**	0.0072	0.0278	−0.0163	1				
**age**	0.0033	0.028	0.0391	−0.0656	1			
**female**	0.0814	0.0338	−0.007	0.0115	−0.2898 *	1		
**urban**	−0.0311	−0.0273	0.0747	−0.0515	0.0065	−0.0422	1	
**group**	−0.0137	−0.038	0.0369	0.056	0.3184 *	−0.0401	0.0561	1

Notes: 1. “screen” variable represents the recreational screen time using. Here it is an ordinal variable (1 = No screen time; 2 = Less than 2 h; 3 = 2 to 3 h; 4 = 3 to 5 h; 5 = more than 5 h). 2. * *p* < 0.05.

**Table A2:** Spearman correlation matrix (recreation screen time as dummy variables).

	_Iscreen_2	_Iscreen_3	_Iscreen_4	_Iscreen_5	Urban	Group	Anxiety	Preanxiety	Midanxiety	Age	Female
**_Iscreen_2**	1										
**_Iscreen_3**	−0.1879 *	1									
**_Iscreen_4**	−0.2208 *	−0.2703 *	1								
**_Iscreen_5**	−0.3340 *	−0.4089 *	−0.4804 *	1							
**urban**	0.0456	0.0085	0.0668	−0.0751	1						
**group**	−0.0503	0.0369	−0.0626	0.0656	0.0561	1					
**anxiety**	0.0359	−0.0286	0.0761	−0.0284	−0.0311	−0.0137	1				
**preanxiety**	−0.0029	−0.0132	0.0683	−0.0112	−0.0273	−0.038	0.6764 *	1			
**midanxiety**	0.0422	−0.0085	0.0657	−0.0452	0.0747	0.0369	0.5908 *	0.7126 *	1		
**age**	0.1157	0.086	−0.1694 *	0.0096	0.0065	0.3184 *	0.0033	0.028	0.0391	1	
**female**	−0.0783	0.0363	0.1459 *	−0.0681	−0.0422	−0.0401	0.0814	0.0338	−0.007	−0.2898	1

Note: (1) _Iscreen_2, _Iscreen3, _Iscreen4, _Iscreen5 represent recreation screen time of “less than 2 h”, “2–3 h”, “3 to 5 h” and “more than 5 h”, respectively. _Iscreen_1 was used as the reference group. (2) * *p* < 0.05.
